# Quantum Computing for Optimal Dispatch of Virtual Power Plants Under Wind and Solar Uncertainty

**DOI:** 10.3390/e28060586

**Published:** 2026-05-25

**Authors:** Ningqiao Liu, Yuxin Zhang, Zhihang Liu, Chao Zheng

**Affiliations:** 1 School of Electrical and Information Engineering, Changsha University of Science and Technology, Changsha 410114, China; 2 School of Energy Storage Science and Engineering, North China University of Technology, Beijing 100144, China; 3 Beijing Key Laboratory of Information Metamaterials, Beijing 100144, China

**Keywords:** quantum computing, coherent ising machine, Virtual Power Plants, wind and solar uncertainty, model predictive control

## Abstract

The modern power system is characterized by large-scale networks, diverse types of sources and loads, and complex grid structures. Virtual Power Plants (VPPs) are proposed to address the operation problem after the integration of Distributed Energy Resources (DERs). Optimization problems in the VPP operation are predominantly mixed-integer programming (MIP) problems belonging to the class of NP-hard problems, motivating the application of quantum computers. Focusing on the VPP optimal dispatch problem under wind and solar uncertainty, we employ the Model Predictive Control (MPC) framework to conduct the VPP intraday rolling dispatch. The classical model and the Quadratic Unconstrained Binary Optimization (QUBO) model for the MPC-based intraday rolling dispatch problem are formulated, respectively. The QUBO formulation of the VPP dispatch problem renders it directly solvable by a specialized quantum computer based on dissipative optical systems: the Coherent Ising Machine (CIM). Compared with the benchmark classical solvers, the experimental results demonstrate the significant computational time reduction capability of CIM. Specifically, compared to Gurobi, Simulated Annealing and Tabu Search, the CIM achieves relative computational time reductions of 75.25%, 99.95% and 99.96%, respectively, while maintaining competitive solution quality. Our work demonstrates the applicability of CIM and its acceleration potential in VPP intraday rolling dispatch, paving the way for the practical application of specialized photonic quantum computers in smart grids.

## 1. Introduction

The power system is transitioning from being dominated by fossil fuel-based generation to a modern power system featuring the joint participation of multiple renewable energy entities [1,2,3]. The modern power system is characterized by large-scale networks, diverse types of sources and loads, and complex grid structures [4,5,6]. Consequently, the computational complexity of problems related to planning and design, operation and dispatch of the power system is increasing rapidly [7,8,9]. For instance, the integration of numerous Distributed Energy Resources (DERs) of varying types into the grid has led to a surge in the number of decision variables and constraints within optimization models [10,11,12,13]. Virtual Power Plants (VPPs) are proposed to address the dispatch and operation problem after the integration of various DERs, such as photovoltaic (PV) systems, wind farms, and battery energy storage system (BESS) [14,15,16]. The core of VPP is its control and coordination center, which possesses functions such as generation management and DG output forecasting [17]. VPP can effectively integrate diverse types of DERs and loads on a large scale, maximize the benefits of distributed generators (DGs), mitigate power fluctuations in the grid, and enhance power supply reliability [18]. This enables VPP to perform functions similar to conventional power plants, participating in electricity market transactions to maximize its economic benefits with optimal dispatch [19]. In particular, integrating large-scale renewable energy resources to smart grids requires addressing the impact of power output uncertainty, which necessitates the rational coordination of BESS and other controllable DGs to achieve stable power output. As a VPP typically consists of uncontrollable DGs, such as wind turbines and PV, and controllable DGs, such as gas turbine and BESS, VPP is well suited for addressing renewable energy generation uncertainty [17]. Optimization problems in VPP operation are predominantly NP-hard mixed-integer programming (MIP) problems [20,21,22]. When the scale of the problem is large, algorithms inevitably fall into the curse of dimensionality, with computation time growing exponentially [23]. Therefore, optimization technologies relying on classical computers might encounter increasing challenges in meeting the operational requirements of the modern power system [24,25,26,27]. Specifically, the time-consuming solution process based on classical computers may hinder timely dispatch, potentially causing the power system to operate in suboptimal economic states for extended periods and even rendering it unable to recover rapidly from faults during extreme events.

A wide range of breakthroughs in the field of quantum technology and quantum computing have been made in recent decades [28,29,30,31,32,33,34,35,36,37], and it has become possible to apply quantum computers to solve optimization problems that are intractable for classical computers [28,38,39]. As an emerging technology, quantum computing executes computational processes by utilizing quantum mechanical properties, such as quantum superposition and quantum entanglement [40]. Compared to classical optimization methods, quantum optimizers show potential advantages in solution efficiency. In the noisy intermediate-scale quantum (NISQ) era of quantum computing [29], compared to universal quantum computers, specialized quantum computers can be more suited for addressing various industrial optimization problems [41,42,43,44,45]. Coherent Ising Machine (CIM) is a kind of specialized quantum computer based on dissipative optical systems for solving combinatorial optimization problems and has been applied to multiple fields [41,46,47,48,49,50,51,52,53,54]. Compared with quantum annealers, such as the superconducting quantum annealers manufactured by D-Wave [45,55], CIM is based on dissipative optical system, operates at room temperature, and features long coherence time and all-to-all connectivity, enabling it to perform better in optimization problem with dense connections [33,55,56,57].

In this work, focusing on the VPP intraday optimal dispatch problem under wind and solar uncertainty, we address the wind and PV generation stochasticity by employing the Model Predictive Control (MPC) framework when conducting the VPP intraday rolling dispatch. The classical model and the Quadratic Unconstrained Binary Optimization (QUBO) model for the MPC-based intraday rolling dispatch problem are formulated, respectively. The QUBO formulation of the MPC-based intraday rolling dispatch problem allows it to be directly solved by CIM. Comparing with the benchmark classical optimization solvers, CIM exhibits significant speedup potential in the exploratory proof-of-concept framework, while maintaining competitive solution quality. By demonstrating the applicability of CIM and its speedup potential in the VPP intraday rolling dispatch problem, our work paves the way for the practical application of CIM in modern power systems.

## 2. Classical Model for MPC-Based Intraday Rolling Dispatch

We consider a VPP consisting of wind turbines, PV, gas turbines and BESS with Time-of-Use (TOU) electricity price taken into consideration. The economic dispatch model of the VPP is described as below. We assume the day-ahead (DA) scheduling of the VPP is predetermined and focus on the intraday rolling dispatch of the VPP. The nomenclature is summarized in Table 1.

A major challenge for the economic dispatch of the VPP is how to address the uncertainty of the wind and PV generation. We employ the Model Predictive Control (MPC) framework [58,59] to conduct the intraday rolling dispatch. MPC has been widely applied to various decision-making problems when facing prediction uncertainty [60,61]. It is a rolling optimization-based decision-making method that can comprehensively consider the system state and the prediction over a finite horizon. For the VPP intraday rolling dispatch problem, the operating day is uniformly divided into *T* periods with each period duration being Δt. At the beginning of each period t=1,2,…,T, the physical states of the gas turbine and the BESS are updated and the ultra-short-term forecasting of wind and PV generation over the prediction horizon $T_{p}$ is acquired. Based on the updated state at each time *t*, the ultra-short-term predictions, and the predetermined DA baselines, the intraday rolling dispatch optimization problem is solved within the MPC framework and an optimal dispatch sequence over the prediction horizon $T_{p}$ is obtained. However, only the dispatch command at time *t* is physically implemented, which will result in the update of the physical states of the gas turbine and the BESS at time t+1. By this rolling cycle of updating, forecasting, optimizing and implementing, the intraday rolling dispatch is iteratively achieved. In this way, the VPP effectively mitigates the uncertainty of wind and PV generation, dynamically tracks the predetermined DA baselines, and maximizes its net revenue.

In the MPC-based intraday rolling dispatch optimization model, the objective function is to maximize the net revenue of the VPP over the prediction horizon $T_{p}$ beginning at time *t*

(1) $$\max\;\sum_{k=t}^{t+T_{p}-1}\left(R_{k}-C_{\mathrm{om},k}-C_{f,k}-C_{\mathrm{pn},k}\right)\;,$$

in which the revenue term $R_{k}$ of the VPP at time step *k* is

(2) $$R_{k}=\rho_{k}^{s}P_{k}\Delta t\;,$$

with

(3) $$P_{k}=P_{k}^{W}+P_{k}^{\mathrm{PV}}+P_{k}^{\mathrm{GT}}+P_{k}^{\mathrm{dis}}-P_{k}^{\mathrm{ch}}\;,$$

where $P_{k}^{W}$ and $P_{k}^{\mathrm{PV}}$ are the ultra-short-term predictions of the wind and PV output. $P_{k}^{\mathrm{GT}}$, $P_{k}^{\mathrm{dis}}$ and $P_{k}^{\mathrm{ch}}$ are the output of the gas turbine, and the discharging and charging power of the BESS, respectively, which are the decision variables to be determined by the MPC-based intraday rolling dispatch optimization. $P_{k}$ is the intraday expected aggregated net power output of the VPP at time step *k*. $\rho_{k}^{s}$ is the TOU electricity price. The cost terms of the VPP include the operation and maintenance (O&M) costs and the penalty cost for deviating from the DA baselines

(4) $$C_{\mathrm{om},k}=M^{\mathrm{GT}}P_{k}^{\mathrm{GT}}\Delta t+M^{\mathrm{bat}}\left(P_{k}^{\mathrm{dis}}+P_{k}^{\mathrm{ch}}\right)\Delta t\;,$$

(5) $$C_{f,k}=\rho_{\mathrm{GT}}P_{k}^{\mathrm{GT}}\Delta t\;,$$

(6) $$C_{\mathrm{pn},k}=\rho_{k}^{\mathrm{pn}}{\left(P_{k}^{*}-P_{k}\right)}^{2}\;,$$

where $C_{\mathrm{om},k}$ denotes the O&M costs of the VPP at time step *k*. $M^{\mathrm{GT}}$ and $M^{\mathrm{bat}}$ represent the O&M costs coefficients of the gas turbine and the BESS, respectively. $C_{f,k}$ and $C_{\mathrm{pn},k}$ represent the fuel cost and the penalty cost of the VPP at time step *k*, with $\rho_{\mathrm{GT}}$ and $\rho_{k}^{\mathrm{pn}}$ denoting the fuel price of the gas turbine and the deviation penalty coefficient. $P_{k}^{*}$ is the DA scheduled power output of the VPP at time step *k*. For the deviation penalty coefficient $\rho_{k}^{\mathrm{pn}}$, we set

(7) $$\rho_{k}^{\mathrm{pn}}=\alpha \frac{\rho_{k}^{s}\Delta t}{P_{\mathrm{unit}}}\;,$$

where $P_{\mathrm{unit}}=1$ MW is introduced for unit consistency. α is the dimensionless penalty multiplier.

The objective function Equation (1) is subject to the following constraints for $\forall k\in \{t,\ldots ,t+T_{p}-1\}$

(8) $$u_{k}^{*}P_{\min}^{\mathrm{GT}}\leq P_{k}^{\mathrm{GT}}\leq u_{k}^{*}P_{\max}^{\mathrm{GT}}\;,$$

where $P_{\max}^{\mathrm{GT}}$ and $P_{\min}^{\mathrm{GT}}$ represent the upper and lower limits of gas turbine output, respectively. $u_{k}^{*}$ is the binary variable indicating the commitment state of the gas turbine. Note that $u_{k}^{*}$ is determined in the DA baselines.

(9) $$-\Gamma_{↓}^{\mathrm{GT}}\Delta t\leq P_{k}^{\mathrm{GT}}-P_{k-1}^{\mathrm{GT}}\leq \Gamma_{↑}^{\mathrm{GT}}\Delta t\;,$$

where $\Gamma_{↓}^{\mathrm{GT}}$ and $\Gamma_{↑}^{\mathrm{GT}}$ are the ramp-down and ramp-up rate limits. For the BESS, we have

(10) $$S_{\min}\leq S_{k}\leq S_{\max}\;,$$

(11) $$S_{k}=S_{k-1}+\frac{\Delta t}{E_{R}}\left(P_{k}^{\mathrm{ch}}\mu_{c}-P_{k}^{\mathrm{dis}}/\mu_{d}\right)\;,$$

(12) $$\delta_{k}^{\mathrm{ch}}+\delta_{k}^{\mathrm{dis}}\leq 1$$

(13) $$0\leq P_{k}^{\mathrm{ch}}\leq \delta_{k}^{\mathrm{ch}}P_{\max}^{\mathrm{ch}}\;,$$

(14) $$0\leq P_{k}^{\mathrm{dis}}\leq \delta_{k}^{\mathrm{dis}}P_{\max}^{\mathrm{dis}}\;,$$

where $S_{\min}$ and $S_{\max}$ are the lower and upper limits of the BESS state of charge (SoC), respectively. $S_{k}$ is the SoC at time step *k*. $E_{R}$ is the rated capacity of the BESS. $\mu_{c}$ and $\mu_{d}$ denote the charging and discharging efficiencies. $\delta_{k}^{\mathrm{ch}}$ and $\delta_{k}^{\mathrm{dis}}$ are binary variables indicating the charging and discharging status of the BESS at time step *k*. Constraint Equation (12) is implemented to prevent simultaneous charging and discharging. $P_{\max}^{\mathrm{ch}}$ and $P_{\max}^{\mathrm{dis}}$ are the maximum charging and discharging power of the BESS. By recursive expansion, Equation (11) can be written as

(15) $$S_{k}=S_{t-1}+\frac{\Delta t}{E_{R}}\sum_{j=t}^{k}\left(P_{j}^{\mathrm{ch}}\mu_{c}-P_{j}^{\mathrm{dis}}/\mu_{d}\right)\;,$$

which will reduce the number of qubits needed to solve the intraday rolling dispatch problem with CIM.

## 3. QUBO Model for MPC-Based Intraday Rolling Dispatch

To solve the MPC-based intraday rolling dispatch problem using CIM, we first formulate the corresponding QUBO model [62]. In general, a QUBO model is used for modeling combinatorial optimization problems, with the basic form being

(16) $$\min\sum_{z_{i},z_{j}\in Z,i\neq j}w_{ij}z_{i}z_{j}+\sum_{z_{i}\in Z}c_{i}z_{i}\;,$$

where $z_{i}$ are binary variables; $Z=\{z_{1},z_{2},\ldots ,z_{n}\}$; and $w_{ij}$ and $c_{i}$ are the quadratic coefficient and the linear coefficient in the QUBO model, respectively. The QUBO model can also be expressed in the matrix form

(17) $$\min z^{⊤}Qz\;,$$

where $z={\left[z_{1},z_{2},\ldots ,z_{n}\right]}^{⊤}$, Q is the QUBO matrix with $Q_{ii}=c_{i}$, and $Q_{ij}=w_{ij}/2$. Given the QUBO matrix Q and via the transformation $s_{i}=2z_{i}-1$, with $s_{i}$ being the Ising spin variable, the QUBO problem can be further converted into an Ising problem, which can be directly solve by CIM [63].

The continuous decision variables need to be discretized by binary variables to be compatible with the CIM hardware. In particular, in the discretization process, the upper and lower limits can be incorporated into it as we show below. For $\forall k\in \{t,\ldots ,t+T_{p}-1\}$, the continuous variables can be written as

(18) $$P_{k}^{\mathrm{GT}}=u_{k}^{*}P_{\min}^{\mathrm{GT}}+u_{k}^{*}\Delta P_{\mathrm{GT}}\sum_{n=0}^{N_{\mathrm{GT}}-1}2^{n}x_{k,n}^{\mathrm{GT}}\;,$$

(19) $$P_{k}^{\mathrm{ch}}=\Delta P_{\mathrm{ch}}\sum_{n=0}^{N_{\mathrm{ch}}-1}2^{n}x_{k,n}^{\mathrm{ch}}\;,$$

(20) $$P_{k}^{\mathrm{dis}}=\Delta P_{\mathrm{dis}}\sum_{n=0}^{N_{\mathrm{dis}}-1}2^{n}x_{k,n}^{\mathrm{dis}}\;,$$

where $x_{k,n}^{\mathrm{GT}},x_{k,n}^{\mathrm{ch}},x_{k,n}^{\mathrm{dis}}\in \left\{0,1\right\}$, $\Delta P_{\mathrm{GT}}=\left(P_{\max}^{\mathrm{GT}}-P_{\min}^{\mathrm{GT}}\right)/\left(2^{N_{\mathrm{GT}}}-1\right)$, $\Delta P_{\mathrm{ch}}=P_{\max}^{\mathrm{ch}}/\left(2^{N_{\mathrm{ch}}}-1\right)$, and $\Delta P_{\mathrm{dis}}=P_{\max}^{\mathrm{dis}}/\left(2^{N_{\mathrm{dis}}}-1\right)$. By introducing non-negative slack variables, other inequality constraints are first converted to equality constraints

(21) $$P_{k}^{\mathrm{GT}}-P_{k-1}^{\mathrm{GT}}+s_{k}^{u}=\Gamma_{↑}^{\mathrm{GT}}\Delta t\;,$$

(22) $$P_{k-1}^{\mathrm{GT}}-P_{k}^{\mathrm{GT}}+s_{k}^{d}=\Gamma_{↓}^{\mathrm{GT}}\Delta t\;,$$

(23) $$S_{k}+s_{k}^{\max}=S_{\max}\;,$$

(24) $$S_{k}-s_{k}^{\min}=S_{\min}\;,$$

where $s_{k}^{u}$, $s_{k}^{d}$, $s_{k}^{\max}$ and $s_{k}^{\min}$ are the slack variables. Those equality constraints are then represented as penalty terms in the QUBO model

(25) $$H_{1}=\lambda_{1}\sum_{k=t}^{t+T_{p}-1}\left[{\left(P_{k}^{\mathrm{GT}}-P_{k-1}^{\mathrm{GT}}+s_{k}^{u}-\Gamma_{↑}^{\mathrm{GT}}\Delta t\right)}^{2}+{\left(P_{k-1}^{\mathrm{GT}}-P_{k}^{\mathrm{GT}}+s_{k}^{d}-\Gamma_{↓}^{\mathrm{GT}}\Delta t\right)}^{2}\right]\;,$$

(26) $$H_{2}=\lambda_{2}\sum_{k=t}^{t+T_{p}-1}\left[{\left(S_{k}+s_{k}^{\max}-S_{\max}\right)}^{2}+{\left(S_{k}-s_{k}^{\min}-S_{\min}\right)}^{2}\right]\;,$$

where $\lambda_{1,2}$ are the QUBO penalty coefficients and $S_{k}$ should be expressed in the form of Equation (15). To deal with the constraint of avoiding simultaneous charging and discharging of the BESS, instead of introducing binary variables $\delta_{k}^{\mathrm{ch}}$ and $\delta_{k}^{\mathrm{dis}}$ and constraint Equation (12) to the QUBO model, which will require a substantial number of qubits, we add a penalty term $H_{3}$ with QUBO penalty coefficient $\lambda_{3}$ to avoid simultaneous charging and discharging

(27) $$H_{3}=\lambda_{3}\sum_{k=t}^{t+T_{p}-1}P_{k}^{\mathrm{ch}}P_{k}^{\mathrm{dis}}\;.$$

With the objective function term $H_{\mathrm{obj}}$ written as

(28) $$H_{\mathrm{obj}}=\sum_{k=t}^{t+T_{p}-1}\left(C_{\mathrm{om},k}+C_{f,k}+C_{\mathrm{pn},k}-R_{k}\right)\;,$$

in which the continuous decision variables $P_{k}^{\mathrm{GT}}$, $P_{k}^{\mathrm{ch}}$ and $P_{k}^{\mathrm{dis}}$ are discretized according to Equations (18)–(20), we now have the MPC-based intraday rolling dispatch problem in its QUBO form

(29) $$\min\;H=H_{\mathrm{obj}}+H_{1}+H_{2}+H_{3}\;.$$

**Figure 1:**
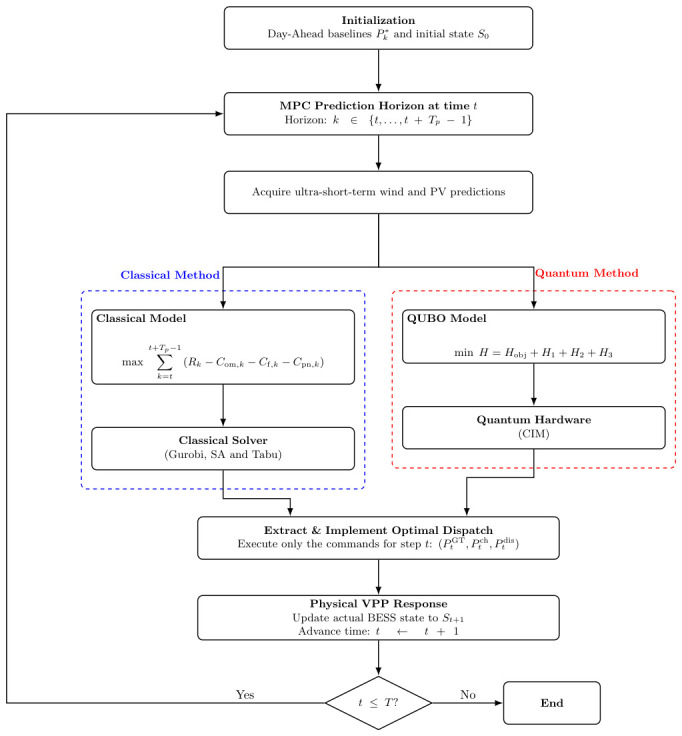
The MPC-based intraday rolling dispatch framework using classical optimization solvers and CIM.

The flowchart of the MPC-based intraday rolling dispatch framework using classical optimization solvers and CIM is presented in Figure 1.

## 4. Experiments

The considered VPP consists of a 30 MW wind farm, a 20 MW PV system, a gas turbine and a BESS. More specifically, the gas turbine has maximum and minimum output limits of 10 MW and 2 MW, respectively, while the BESS operates with a rated capacity of $E_{R}=10$ MWh and a maximum charge/discharge power limit of $P_{\max}^{\mathrm{ch}}=P_{\max}^{\mathrm{dis}}=5$ MW. The TOU electricity prices for valley (23:00–7:00), flat (07:00–10:00, 15:00–18:00), and peak (10:00–15:00, 18:00–23:00) periods are 0.3, 0.6, and 1.0 CNY/kWh, respectively.

Wind turbine and PV output are highly stochastic, and their nonlinear outputs will change with factors such as time and environment [64,65,66,67]. The wind farm output is basically determined by wind speed, and the specific relationship can be modeled as

(30) $$P^{W}=\left\{\begin{array}{cc} 0, & u\leq u_{\mathrm{in}}\\ \alpha_{w}u^{3}-\beta_{w}P_{R}^{W}, & u_{\mathrm{in}}<u\leq u_{R}\\ P_{R}^{W}, & u_{R}<u\leq u_{\mathrm{out}}\\ 0, & u>u_{\mathrm{out}} \end{array}\right.\;,$$

with

(31) $$\left\{\begin{array}{c} \alpha_{w}=P_{R}^{W}/\left(u_{R}^{3}-u_{\mathrm{in}}^{3}\right)\\ \beta_{w}=u_{\mathrm{in}}^{3}/\left(u_{R}^{3}-u_{\mathrm{in}}^{3}\right) \end{array}\right.\;,$$

where $P^{W}$ is the wind farm output. $P_{R}^{W}$, *u* and $u_{R}$ represent the rated power, actual wind speed and the rated wind speed, respectively. $u_{\mathrm{in}}$ and $u_{\mathrm{out}}$ are the cut-in and cut-out wind speeds. $\alpha_{w}$ and $\beta_{w}$ are fitting coefficients determined by the physical characteristics of the wind turbine.

**Figure 2:**
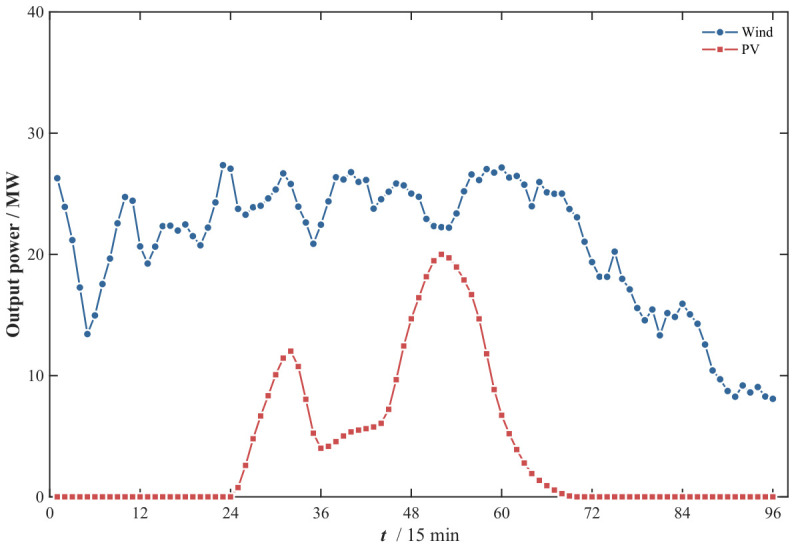
DA forecast of wind and solar output.

The stochastic nature of PV output is reflected in its dependence on solar irradiance and ambient temperature. The PV output can be expressed as

(32) $$P^{\mathrm{PV}}=P_{\mathrm{ref}}^{\mathrm{PV}}\frac{G}{G_{\mathrm{ref}}}\left[1+\lambda_{T}\left(\theta_{c}-\theta_{\mathrm{ref}}\right)\right]\;,$$

where $P^{\mathrm{PV}}$ is the PV output power; $P_{\mathrm{ref}}^{\mathrm{PV}}$ is the rated output power under standard reference conditions (solar irradiance $G_{\mathrm{ref}}=1\;\mathrm{kW}/m^{2}$, reference temperature $\theta_{\mathrm{ref}}=298.15\;K$). *G* and $\lambda_{T}$ represent the predicted solar irradiance and the power temperature coefficient, respectively. $\theta_{c}$ denotes the operating temperature of the PV cells. The DA wind and PV output predictions are presented in Figure 2. In general, the DA scheduling can be determined by scenario-based stochastic programming or robust optimization [68,69]. In this work, as we focus on the intraday rolling dispatch problem, the DA baselines are determined by a rule-based approach considering TOU electricity price and DA wind and PV output predictions.

### 4.1. The CIM Setup

The employed CIM is a photonic specialized quantum computer featuring advantages such as operating at room temperature and full connectivity between qubits [33]. The CIM utilizes a femtosecond fiber pulsed laser to generate optical pulses and employs the erbium-doped fiber amplifier (EDFA) to achieve power amplification. The frequency of the optical pulse is then doubled through a periodically poled lithium niobate (PPLN1) crystal. Subsequently, the frequency-doubled optical pulse is converted into the signal light by the PPLN2 crystal and forms degenerate optical parametric oscillators (DOPO) within the fiber cavity. This process generates optical pulses with specific phases and amplitudes, which can serve as photonic qubits. All photonic qubits are stored within the fiber loop for subsequent evolution.

**Figure 3:**
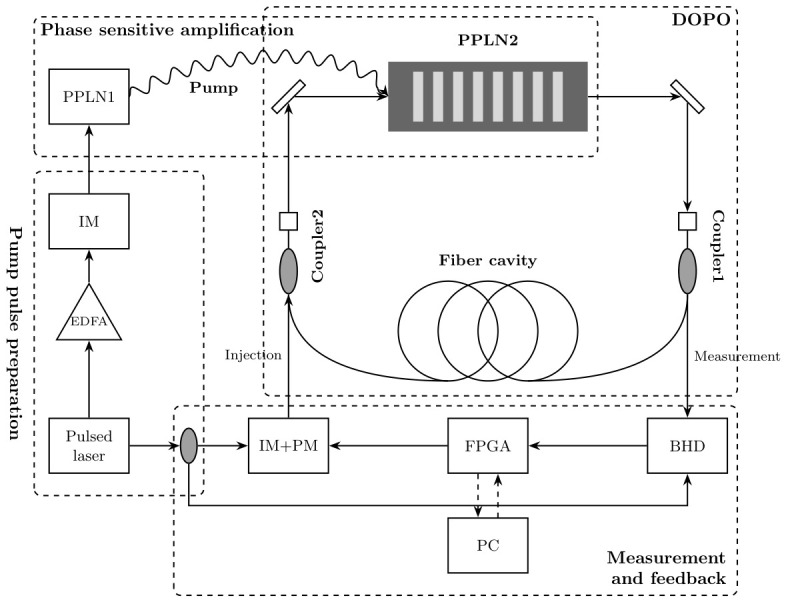
Architecture of the employed CIM.

The measurement and feedback module is composed of balanced homodyne detection (BHD), a phase modulator (PM), an intensity modulator (IM), a field-programmable gate array (FPGA) and a host computer (PC). By controlling the host computer, the matrix of the Ising problem to be solved is downloaded to the FPGA. The FPGA measures the amplitude of the optical pulses in the fiber loop via the BHD. Based on the target Ising matrix, the FPGA calculates a feedback signal and modulates the feedback optical pulse through the IM and PM. By utilizing the mutual interference between the feedback optical pulse and the optical pulses circulating in the fiber loop, the photonic qubits in the loop are guided to evolve toward the lowest Hamiltonian value of the Ising problem and the final solution to the Ising problem is encoded in the phase information of the photonic qubits. For the considered VPP dispatch problem, each continuous variable is encoded with 5 qubits and the slack variables are encoded with 4 or 5 qubits. In summary, 528 qubits are required for the problem, while the CIM employed consists of 550 qubits. The architecture of the employed CIM is illustrated in Figure 3.

### 4.2. Results and Analysis

The MPC-based intraday rolling dispatch optimization problem is solved using the proposed CIM alongside three benchmark classical methods: the Gurobi optimizer, Simulated Annealing (SA) [70], and Tabu Search (TS) [71]. Gurobi is at the default setting, with MIPGap being 0.01%. For the SA algorithm, the initial temperature is set to 5000, the minimum termination temperature to 0.1, the cooling rate to 0.95, and the Markov chain length at each temperature gradient to 50. For the TS algorithm, the maximum number of iterations is set to 150. For each iteration, the neighborhood size is set to 100 and the maximum length of the tabu list is set to 30. The configurations of SA and TS are selected based on extensive simulations with the goal of achieving a competitive solution quality. All the classical methods experiments are coded in Python 3.10 and conducted with an Intel Core i7-12700H CPU. For each solution method, the experiment was executed 30 times, and the performance results are presented in Table 2.

**Figure 4:**
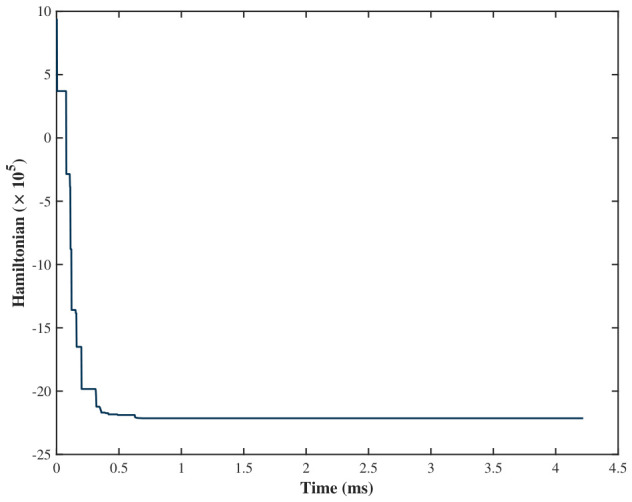
Evolution of the Ising Hamiltonian for the MPC-based intraday rolling dispatch problem solved on the CIM.

**Figure 5:**
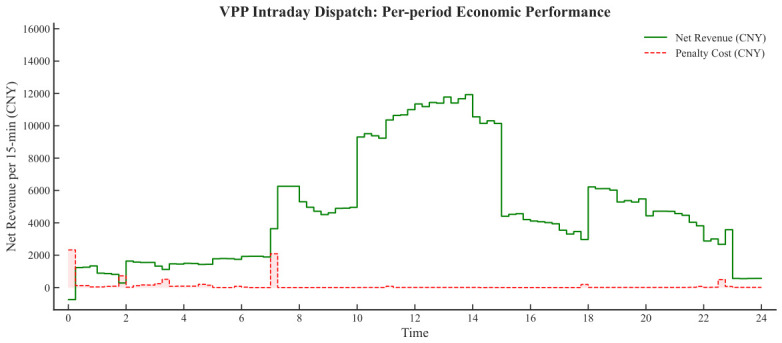
Per-period net revenue and penalty cost under the optimal dispatch of CIM.

**Figure 6:**
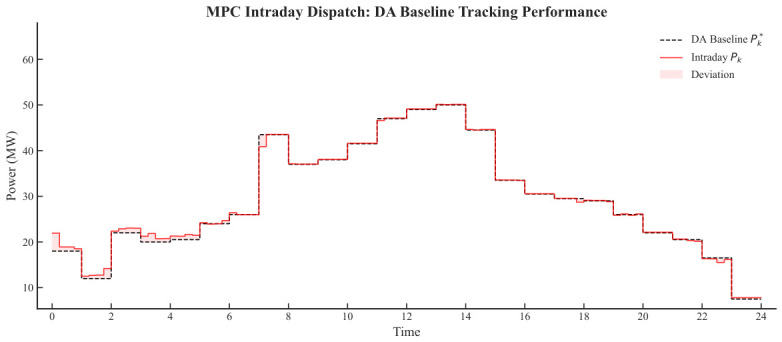
DA baseline tracking under the optimal dispatch of CIM.

**Figure 7:**
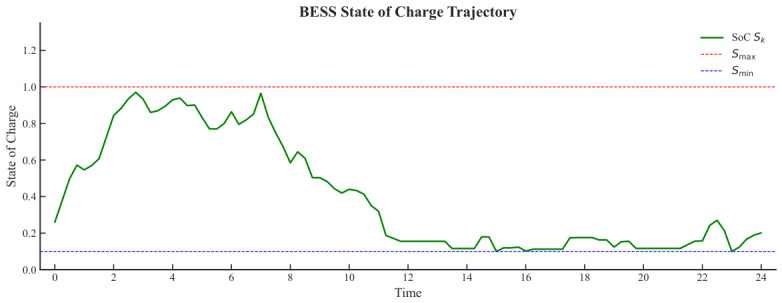
SoC of the BESS under the optimal dispatch of CIM.

**Figure 8:**
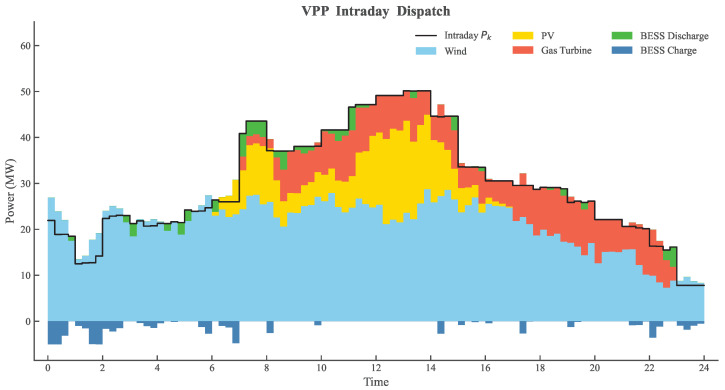
VPP intraday dispatch details under the optimal dispatch of CIM.

We define the relative time reduction $\eta =\frac{T_{C}-T_{\mathrm{CIM}}}{T_{C}}$ to quantify the acceleration capability of the CIM, with $T_{C}$ and $T_{\mathrm{CIM}}$ representing the Time-to-Solution (TTS) for the benchmark classical methods and the CIM, respectively. For the MPC-based intraday rolling dispatch problem, the optimization is executed 96 times in the operating day and the TTS means the computation time for a single dispatch instance. Compared to the Gurobi optimizer, SA and TS, the CIM achieves relative time reduction η of 75.25%, 99.95% and 99.96%, respectively, demonstrating its significant acceleration capability. In detail, the mean TTS of the CIM is 1.14 ms, with the standard deviation (SD) of 0.48 ms. Figure 4 illustrates the evolution of the Ising Hamiltonian for the MPC-based intraday rolling dispatch problem, showing a rapid convergence to the lowest energy state within 1 ms. In contrast, to achieve a competitive solution quality, the mean TTS values of SA and TS are 2210.89 ms and 3181.35 ms, respectively. In terms of intraday net revenue, the optimal solution found by Gurobi is 456,861 CNY, and the mean optimality gaps of CIM, SA and TS are 0.23%, 0.27% and 0.25%, respectively, demonstrating the highly competitive performance of the CIM. Under the optimal intraday dispatch of CIM, we present the net revenue and penalty cost at each period *t* in Figure 5, the DA baseline tracking performance in Figure 6, SoC dynamics in Figure 7, and VPP dispatch details in Figure 8.

More specifically, the green solid line and the red dashed line in Figure 5 represent the net revenue and penalty cost at each period *t* under the optimal dispatch of CIM. The per-period net revenue is much larger around 10:00–15:00 than around 0:00–7:00, which are the off-peak hours of the TOU electricity price. In most of the 96 periods, the penalty cost is close to zero, reflecting the regulation capability of the BESS and the gas turbine. However, around 0:00, 2:00, and 7:00, the penalty cost is significant, reflecting the stochasticity of the wind and PV output, which is out of the regulation capability of the BESS and the gas turbine. The per-period net revenue and penalty cost performance can be further explained by the DA baseline tracking performance in Figure 6. The intraday expected aggregated net power output of the VPP $P_{k}$, represented as the red solid line, has similar peak periods to the per-period net revenue. The intraday $P_{k}$ deviation from the DA baseline $P_{k}^{*}$ shows strict correspondence to the per-period penalty cost as expected.

The BESS plays a critical role in tracking the DA baseline, as it possesses bidirectional regulation capability. In the off-peak hours of the TOU electricity price, the BESS is expected to charge and the SoC dynamics manifest as an overall increasing trajectory, which is consistent with the observations in Figure 7. From 0:00 to 7:00, SoC rapidly increases and approaches the upper capacity limit around 3:00. Subsequently, Soc fluctuations at a high level until it approaches the upper capacity limit again around 7:00. In the morning peak periods, the BESS discharges and approaches the lower capacity limit around 12:00. Since the period of 12:00–22:00 is the peak of TOU electricity price, the BESS is discouraged from charging and SoC manifests minor fluctuations in response to the wind and PV output stochasticity until 22:00. The gas turbine serving as the other flexible regulation resource is instrumental in addressing the uncertainty of wind and PV generation. As we show in Figure 8, between 8:00 and 23:00, the output of the gas turbine changes dynamically between every period *t*, indicating the essential role it plays in tracking the DA baseline. In particular, between 12:00 and 22:00, the gas turbine plays a more active and major role than BESS in addressing the uncertainty of wind and PV generation.

## 5. Discussion and Conclusions

The modern power system features the participation of diverse types of DERs and loads on a large scale. While VPP is effective in addressing the operation problem after the integration of various DERs, optimization problems in the VPP operation are NP-hard MIP problems. In this work, we focused on the VPP intraday dispatch problem under wind and solar uncertainty, which necessitates the rational coordination of BESS, gas turbines, and other controllable DGs to achieve the scheduled power output. By employing the MPC framework to conduct the VPP intraday rolling dispatch, we successfully addressed the uncertainty of wind and PV generation. The MPC-based intraday rolling dispatch problem was formulated as classical and QUBO models, respectively. The QUBO formulation of the VPP dispatch problem renders it directly solvable by the CIM. We compared the computation performance of the CIM to three benchmark classical solvers: Gurobi, SA and Tabu. While maintaining competitive solution quality, the results demonstrate the significant computational time reduction capability of CIM, which demonstrates the applicability of CIM and its acceleration potential in VPP intraday dispatch. Our work paves the way for practically applying specialized quantum computers in modern power system facing renewable energy uncertainty. However, we must clarify that, as an exploratory proof-of-concept investigation of applying CIM to VPP operations, the observed improvements should be understood as instance-specific gains. More advanced or specialized classical approaches that are commonly used in VPP optimization such as Benders decomposition may achieve better computational performance in terms of solving time. Based on the experimental investigation of performance of the CIM [55] and the all-to-all connectivity of CIM, the CIM-based approaches are expected to offer meaningful benefits over classical methods when facing optimization problems characterized by strong global coupling. Currently, limited by the number of available qubits, the size of the problem that can be addressed by the CIM is at the intermediate scale. Nevertheless, the design of CIM is based on the minimum gain principle and the crucial evolution time does not scale with the problem size [72]. When discretizing continuous decision variables into binary form for the QUBO formulation, the number of qubits used for each variable generally affects the solution accuracy and modeling fidelity. In principle, higher discretization resolution leads to higher solution accuracy and modeling fidelity. Along with the hardware development of CIM, this should be a important problem to investigate. In this work, the MPC with point forecasts provides a tractable baseline. Extending the framework to more advanced uncertainty models such as stochastic programming requires further investigation. Moreover, real-world VPP operation often involves additional complexities such as network constraints and market participation. Therefore, the applicability of the CIM method may rely on its integration into a hybrid quantum-classical computing architecture. The overall problem structure and nonlinearities can be managed by classical computing, whereas the CIM provides the specialized hardware speedup for subproblems.

## Figures and Tables

**Table 1:** Nomenclature.

**Indices and Sets**	
*t*	Index for the time period of the operating day
*T*	Total number of periods in the operating day
*k*	Time step index within the prediction horizon
$T_{p}$	Prediction horizon
Δt	Duration of each period
Z	Set of binary variables in the QUBO model
i,j	Indices for the QUBO binary variables
**Parameters and Constants**	
$P_{k}^{W},P_{k}^{\mathrm{PV}}$	Ultra-short-term predictions of wind and PV generation
$P_{k}^{*}$	Day-ahead (DA) scheduled power output of the VPP (MW)
$\rho_{k}^{s}$	Time-of-Use (TOU) electricity price (CNY/kWh)
$M^{\mathrm{GT}},M^{\mathrm{bat}}$	Operation and maintenance (O&M) cost coefficients (CNY/kWh)
$\rho_{\mathrm{GT}}$	Fuel price of the gas turbine (CNY/kWh)
$\rho_{k}^{\mathrm{pn}}$	Deviation penalty coefficient
α	Dimensionless penalty multiplier
$u_{k}^{*}$	Binary variable indicating DA commitment state of the gas turbine
$P_{\max}^{\mathrm{GT}},P_{\min}^{\mathrm{GT}}$	Upper and lower limits of gas turbine output (MW)
$\Gamma_{↑}^{\mathrm{GT}},\Gamma_{↓}^{\mathrm{GT}}$	Ramp-up and ramp-down rate limits of the gas turbine
$S_{\max},S_{\min}$	Upper and lower limits of the BESS state of charge (SoC)
$E_{R}$	Rated capacity of the BESS
$\mu_{c},\mu_{d}$	Charging and discharging efficiencies of the BESS
$P_{\max}^{\mathrm{ch}},P_{\max}^{\mathrm{dis}}$	Maximum charging and discharging power of the BESS (MW)
$N_{\mathrm{GT}},N_{\mathrm{ch}},N_{\mathrm{dis}}$	Number of qubits for discretizing continuous power variables
$\lambda_{1},\lambda_{2},\lambda_{3}$	Penalty coefficients in the QUBO model
**Variables**	
$P_{k}^{\mathrm{GT}}$	Output of the gas turbine (MW)
$P_{k}^{\mathrm{ch}},P_{k}^{\mathrm{dis}}$	Charging and discharging power of the BESS (MW)
$P_{k}$	Intraday expected aggregated net power output of the VPP (MW)
$S_{k}$	SoC of the BESS
$\delta_{k}^{\mathrm{ch}},\delta_{k}^{\mathrm{dis}}$	Binary variables indicating charging and discharging status
$R_{k}$	Revenue of the VPP
$C_{\mathrm{om},k}$	O&M costs of the VPP
$C_{f,k}$	Fuel cost of the VPP
$C_{\mathrm{pn},k}$	Penalty cost for deviating from the DA baselines

**Table 2:** Comparison of Gurobi, CIM, SA, and Tabu.

	Gurobi	CIM	SA	Tabu
Mean TTS ± SD (ms)	4.61±5.69	1.14±0.48	2210.89±538.65	3181.35±700.72
Relative time reduction (%)	75.25	−	99.95	99.96
Mean net revenue ± SD	456,861 ± 0	455,792 ± 266	455,635 ± 551	455,740 ± 645
Optimality gap (%)	−	0.23	0.27	0.25
Mean penalty cost ± SD	8358±0	8883±192	9042±429	8931±537

## Data Availability

The datasets generated and analyzed for the this work are available from the corresponding author upon reasonable request.

## Abbreviations

The following abbreviations are used in this manuscript:

CIM	Coherent Ising Machine
VPPs	Virtual Power Plants
QUBO	Quadratic Unconstrained Binary Optimization
MPC	Model Predictive Control
DERs	Distributed Energy Resources
TOU	Time-of-Use
SA	Simulated Annealing
TS	Tabu Search
TTS	Time-to-Solution
